# Alkhurma haemorrhagic fever virus causes lethal disease in IFNAR^-/-^ mice

**DOI:** 10.1080/22221751.2021.1932609

**Published:** 2021-06-06

**Authors:** Bharti Bhatia, Elaine Haddock, Carl Shaia, Rebecca Rosenke, Kimberly Meade-White, Amanda J. Griffin, Andrea Marzi, Heinz Feldmann

**Affiliations:** a Laboratory of Virology, National Institute of Allergy and Infectious Diseases, National Institutes of Health, Rocky Mountain Laboratories, Hamilton, MT, USA; b Rocky Mountain Veterinary Branch, Division of Intramural Research, National Institute of Allergy and Infectious Diseases, National Institutes of Health, Rocky Mountain Laboratories, Hamilton, MT, USA

**Keywords:** Alkhurma haemorrhagic fever virus, AHFV, flavivirus, mouse model, pathogenesis

## Abstract

Alkhurma haemorrhagic fever virus (AHFV), a tick-borne flavivirus closely related to Kyasanur Forest disease virus, is the causative agent of a severe, sometimes fatal haemorrhagic/encephalitic disease in humans. To date, there are no specific treatments or vaccines available to combat AHFV infections. A challenge for the development of countermeasures is the absence of a reliable AHFV animal disease model for efficacy testing. Here, we used mice lacking the type I interferon (IFN) receptor (IFNAR^-/-^). AHFV strains Zaki-2 and 2003 both caused uniform lethality in these mice after intraperitoneal injection, but strain 2003 seemed more virulent with a median lethal dose of 0.4 median tissue culture infectious doses (TCID_50_). Disease manifestation in this animal model was similar to case reports of severe human AHFV infections with early generalized signs leading to haemorrhagic and neurologic complications. AHFV infection resulted in early high viremia followed by high viral loads (<10^8^ TCID_50_/g tissue) in all analyzed organs. Despite systemic viral replication, virus-induced pathology was mainly found in the spleen, lymph nodes, liver and heart. This uniformly lethal AHFV disease model will be instrumental for pathogenesis studies and countermeasure development against this neglected zoonotic pathogen.

## Introduction

Alkhurma haemorrhagic fever virus (AHFV) is an enveloped, positive-sense RNA virus belonging to the family *Flaviviridae* [1,2]. It is a variant of Kyasanur Forest disease virus (KFDV) and causes severe haemorrhagic/encephalitic disease in humans (AHF) with a case fatality rate of 1%–20% [3]. AHFV was first isolated in Jeddah, Saudi Arabia, in the 1990s from the blood of a butcher suffering from a haemorrhagic fever syndrome [4]. Since then, several hundred cases of AHF have been reported from Saudi Arabia and Egypt [5]. The geographic distribution of the virus is likely broader with cases being underreported.

*Ornithodoros savignyi* and *Hyalomma dromedarii* are the main tick vectors responsible for maintaining and transmitting AHFV [6,7]. Livestock, mainly camels and sheep, seem to be involved in AHFV amplification and transmission between ticks [8,9]. Humans acquire AHFV through tick bites or contact with blood of viremic livestock; human-to-human transmission has not been reported [3,10,11].

The initial symptoms of an AHFV infection may include fever, headache, diarrhoea, vomiting, muscle and joint pain, a loss of appetite and chills [5,12,13]. Some patients display neurologic and haemorrhagic symptoms and multi-organ failure that precedes fatal outcomes. Hospitalized patients show elevated liver enzymes, leukopenia, proteinuria and thrombocytopenia [9,13,14]. Currently, there are no specific treatments or vaccines available for AHFV [15]. Animal disease models for AHFV have so far been restricted to immunocompetent laboratory mice that display variable disease outcomes from asymptomatic to mild disease to partial lethality [16,17].

To establish a uniformly lethal mouse model, we utilized mice lacking the type I interferon (IFN) receptor (IFNAR^-/-^). Due to their immunocompromised status, these mice are generally more susceptible to infections and have been previously used to establish mouse disease models for a wide range of viruses such as Zika virus (ZIKV), Dengue virus (DENV), Yellow Fever virus (YFV), West Nile virus (WNV), Lassa virus and Crimean Congo Haemorrhagic Fever virus (CCHFV) [18–23]. Here, we show that infection with the Zaki-2 and 2003 strains of AHFV resulted in uniformly lethal disease in a dose-dependent manner. AHFV strain 2003 was more virulent with a median lethal dose (LD_50_) of 0.4 median tissue culture infectious dose (TCID_50_). AHFV infection resulted in early uncharacteristic disease signs followed by haemorrhagic and neurologic manifestations. Infection was systemic starting with early high viremia (up to 10^7^ TCID_50_/ml) followed by high viral organ loads (up to 10^8^ TCID_50_/g tissue). Despite systemic virus replication, pathology was mainly found in the liver, spleen, heart and lymph nodes. In conclusion, IFNAR^-/-^ mice present a uniformly lethal AHFV disease model that can provide valuable insights into AHFV pathogenesis and will be instrumental in vaccine development.

## Materials and methods

### Ethics statement

All infectious *in vitro* and *in vivo* work with AHFV was performed in the BSL-4 laboratory of the Rocky Mountain Laboratories (RML), Division of Intramural Research (DIR), National Institute of Allergy and Infectious Disease (NIAID), National Institutes of Health (NIH) using standard operating protocols (SOPs) approved by the RML Institutional Biosafety Committee (IBC). Animal work was approved by the RML Institutional Animal Care and Use Committee (IACUC). All animal procedures were carried out by trained and certified personnel in accordance with the guidelines of the Association for Assessment and Accreditation of Laboratory Animal Care, International and the Office of Laboratory Animal Welfare. Mice were group housed in HEPA-filtered cage systems enriched with nesting material. Commercial food and water were available *ad libitum*. Humane endpoint criteria in compliance with IACUC-approved scoring parameters were used to determine when animals should be humanely euthanized.

### Cells and viruses

Vero cells (African green monkey kidney origin) were grown at 37°C and 5% CO_2_ in Dulbecco’s modified Eagle’s medium (DMEM) (Sigma-Aldrich) containing 2%–10% fetal bovine serum (FBS) (Wisent Inc.), 2 mM L-glutamine (Thermo Fisher Scientific), 50 U/mL penicillin (Thermo Fisher Scientific), and 50 μg/mL streptomycin (Thermo Fisher Scientific). AHFV, strain Zaki-2 (GenBank Accession No. JF416957; passage: suckling mouse brain (SMB) + 1, mouse brain+2, Vero+4 and VeroE6 + 1) is the prototype strain isolated in 1994 from a human in Jeddah, Saudi Arabia [4]. AHFV, strain 2003 (GenBank Accession No. JF416954; passage: SMB+1, VeroE6 + 2) was isolated from a human from Makkah, Saudi Arabia [24]. Both strains were kindly provided by the Viral Special Pathogens Branch, Centers for Disease Control and Prevention, Atlanta, Georgia, United States. Both AHFV strains were passaged one more time on VeroE6 cells. Sequencing confirmed a lack of mutations compared to GenBank entries.

### Mouse studies

IFNAR^-/-^ mice (C57BL/6 background) were obtained from an in-house breeding colony. In total, we performed three studies. Initially (pilot study), six mice per group (female/male) were infected intraperitoneally (IP) with 10 or 1000 TCID_50_ of AHFV, strain Zaki-2 and strain 2003 (2 sites, 0.1 ml each). Subsequently, the LD_50_ of AHFV strain 2003 was determined using groups of six mice (female/male). The mice were infected IP with 0.01, 0.1, 1, or 10 TCID_50_ of AHFV (2 sites, 0.1 ml each). For the final pathogenesis study, 18 mice (female/male) were infected IP with 1000 LD_50_ (400 TCID_50_) of AHFV strain 2003 (2 sites, 0.1 ml each). On days 2 and 4, six mice were euthanized and necropsied for organ collection and six mice were observed for survival. Samples were also collected from four uninfected control mice. In all studies, mice were monitored for clinical signs until predetermined euthanasia, humane endpoint or study endpoint at 42 days post infection (dpi). Prior to euthanasia, a single, terminal blood sample was collected from deeply anesthetized animals by intracardiac puncture.

### AHFV titrations

Mouse tissue samples were homogenized in 1 ml of plain DMEM with a stainless-steel bead at 30 Hz for 10 min using a Tissue Lyser II (Qiagen). The clear homogenate was separated from tissue debris by centrifugation at 8000 rpm for 10 min. Serial dilutions (10-fold) of tissue homogenate were prepared in DMEM and used to inoculate confluent monolayers of VeroE6 cells in triplicates. The cytopathic effect was monitored until at least 96 h post-inoculation and the TCID_50_ was calculated for each sample employing the Reed and Muench method [25].

### Haematology and blood chemistry

Whole blood was collected into EDTA-coated tubes. Complete blood counts (CBCs) were performed from EDTA-treated blood using the Haematrue blood analyzer (HESKA). Blood chemistry profiles were obtained from serum samples using the Piccolo point of care chemistry analyzer (Abaxis).

### Histopathology and in-situ hybridization (ISH)

Tissue specimens (<30 mg) were fixed by immersion in 10% neutral buffered formalin for a minimum of 7 days prior to removal from biocontainment according to SOP approved by the IBC. Tissues were processed with a Sakura VIP-6 Tissue Tek on a 12-h automated schedule using a graded series of ethanol, xylene and paraffin. Embedded tissues were sectioned at approximately 4 micrometers, dried overnight at 42°C and stained with haematoxylin and eosin (H&E) for histological examination by a board-certified veterinary pathologist. Chromogenic detection of Alkhumra viral RNA was performed using the RNAscope VS Universal AP assay (Advanced Cell Diagnostics Inc.) on the Ventana Discovery ULTRA stainer using a probe targeting the AHFV genome sequence at position 7597-8486 (Advanced Cell Diagnostics Inc. cat#591199). ISH was performed according to manufacturer’s instructions.

### Quantification of cytokines in plasma

Cytokine levels in plasma derived from EDTA-treated blood of infected mice were analyzed using a ProcartaPlex^TM^ Multiplex Immunoassay kit (Invitrogen). Briefly, 25 µL of irradiated (10 megarads [26]) plasma was run in duplicate with the cytokine assay kit as per manufacturer’s instructions. Samples were then run on the Bio-Luminex 200 (Bio-Rad) and analyzed using the Bio-Plex Manager software version 6.0. Sample groups were compared by 1-way analysis of variance (ANOVA) with Tukey post-test using Prism 7 (GraphPad).

### Enzyme-linked immunosorbent assay (ELISA)

ELISA antigen was prepared by infecting VeroE6 cells with AHFV strain 2003 and collecting supernatant at 48 h post infection. Following low-speed centrifugation, AHFV was pelleted by ultracentrifugation, resuspended in PBS + 2% Triton-X 100 and irradiated with 10 megarads [26]. ELISA plates (96-well flat bottom, maxisorp, NUNC, Waltham, MA, USA) were coated with 100 µl of the antigens (1:1000 dilution in PBS) at 4°C overnight and blocked for 1 h at room temperature with 5% powdered milk in PBS and 0.05% Tween 20 (Fisher Scientific) (PBST). Subsequently, serial dilutions of mouse sera in PBST were added to the plate and incubated for 1 h at room temperature. Convalescent serum from mice infected with KFDV was used as a positive control. Detection was performed using anti-mouse IgG coupled with horse radish peroxidase (Jackson ImmunoResearch) for 1 h at room temperature followed by ABTS substrate solution (Seracare) for 15 min at room temperature. Plates were read at 405 nm using an ELISA reader (BioTek Instruments).

### Statistical analysis

All statistical analysis was performed in Prism 7 (GraphPad). Animal body weight, cytokine, blood chemistry and haematology data were evaluated using unpaired *t* test to evaluate statistical significance. Survival curves were examined for statistical significance using the Mantel–Cox test. Differences in time to death were evaluated using the Mann–Whitney test. Statistically significant differences are indicated as follows: ****p < 0.0001, ***p < 0.001, **p < 0.01 and *p < 0.05.

## Results

Our goal was to establish an easily manageable, very reliable, highly susceptible, and uniformly lethal AHFV mouse model for countermeasure development. Even though intradermal or subcutaneous routes of infection may better mimic natural infection through tick bite, we preferred the IP route for delivery as it is very consistent and well characterized in mouse models [27].

### AHFV caused uniformly lethal infection in IFNAR^-/-^ mice

**Figure 1.**
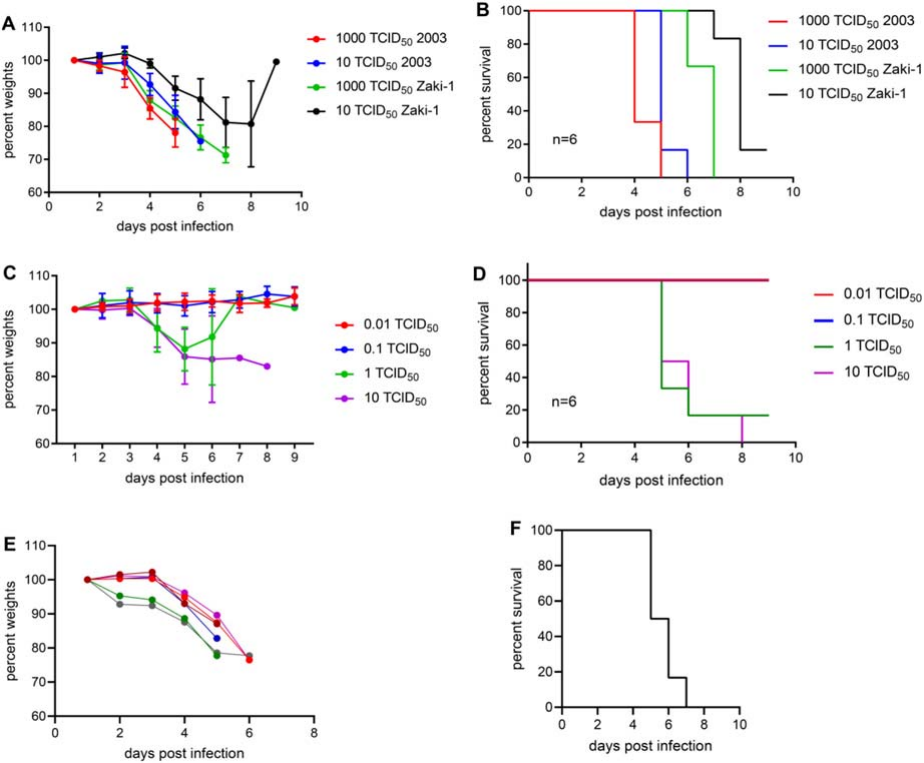
AHFV infection and LD_50_ determination in IFNAR^-/-^ mice. For the pilot study, groups of six mice were infected IP with 10 or 1000 TCID_50_ of either AHFV, strain 2003 or AHFV, strain Zaki-2. (A) Body weight and (B) survival curves are shown. Disease progression was significantly advanced for strain 2003 with both challenge doses (*p* = 0.0022). For the determination of the LD_50_, groups of six mice were infected with increasing doses (0.01–10 TCID_50_) of AHFV strain 2003 via the IP route. (C) Body weight (note, green line represents a single survivor from 6 dpi on) and (D) survival curves are shown. For the pathogenesis study, groups of six mice were infected with 1000 LD_50_ of AHFV 2003 (400 TCID_50_ per animal) via the IP route. (E) Body weight (each line represents one mouse) and (F) survival curves are shown. Error bars represent standard deviation.

First, we performed a pilot experiment to determine disease manifestation and progression in IFNAR^-/-^ mice. We used two AHFV strains, Zaki-2 and 2003. Groups of six mice were infected via the IP route with either 10 or 1000 TCID_50_ of AHFV. Mice started to show first clinical signs at 3 dpi including weight loss, hunched posture, ruffled fur and lethargy (Figure 1(A)). Animals progressed over the next 2–5 days to neurological signs such as ataxia, hind limb paralysis and tremors. When animals reached the humane endpoint between 5 and 8 dpi they were euthanized. The clinical manifestation and outcome of infection were similar with both strains, Zaki-2 and 2003, albeit disease progression was significantly advanced with both challenge doses of strain 2003 (*p* = 0.0022) leading to earlier euthanasia (Figure 1(B)). With both strains, clinical progression and infection outcomes were dose dependent. A uniformly lethal outcome was achieved for strain 2003 at both doses, whereas strain Zaki-2 caused 100% and 83.3% lethality at the higher and lower dose, respectively (Figure 1(B)).

As AHFV strain 2003 was more virulent, we continued model development with this strain. Next, we determined the LD_50_ in IFNAR^-/-^ mice. Groups of six mice were injected IP with 0.01, 0.1, 1, or 10 TCID_50_ of AHFV 2003. Mice were monitored for disease progression and euthanized at the humane endpoint. The LD_50_ was calculated to be 0.4 TCID_50_ (Figure 1(C,D)**)**. None of the surviving mice seroconverted as analyzed by an AHFV-specific IgG ELISA (data not shown) indicating that the animals were not infected at the lower challenge doses.

### AHFV infection of IFNAR^-/-^ mice resulted in blood alterations and a systemic inflammatory response

**Figure 2.**
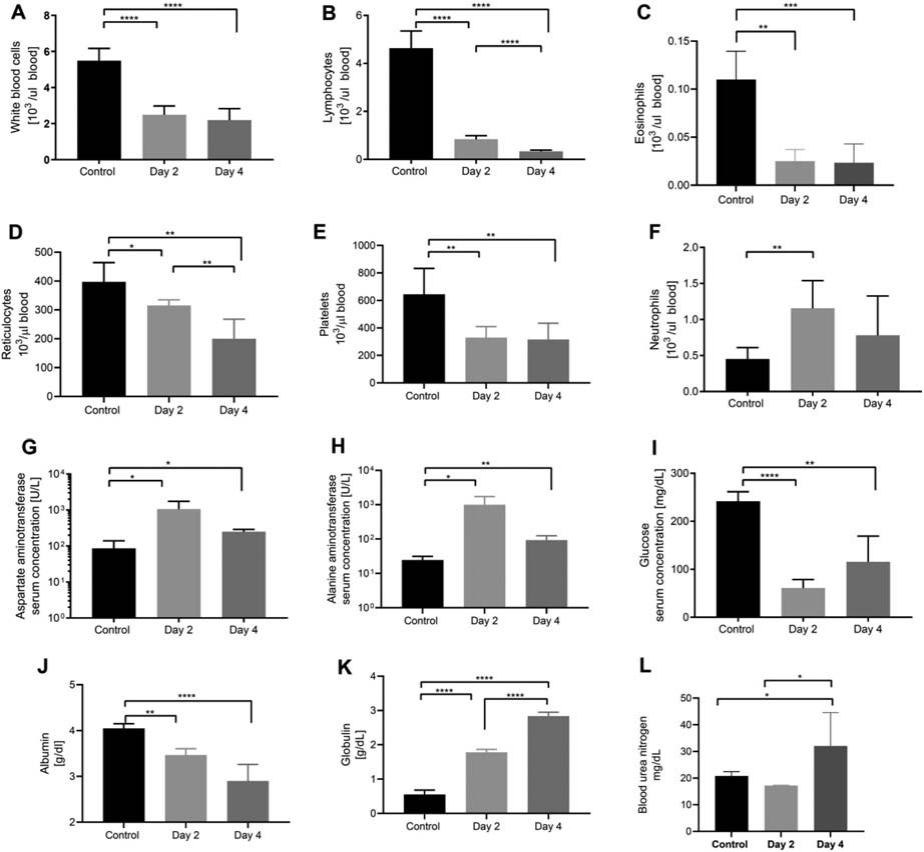
Haematology and blood chemistry in IFNAR^-/-^ mice infected with AHFV. Groups of six mice were infected with 1000 LD_50_ of AHFV 2003 (400 TCID_50_ per animal) via the IP route. On 2 and 4 dpi samples were collected from mice and CBCs and blood chemistry were performed and compared to mock-infected animals. (A–F) Selected haematology cell counts are displayed. (G–L) Selected blood chemistry parameters are displayed. Due to poor sample quality, graphs G–L are based on three samples from 4 dpi group. Error bars represent standard deviations. Statistical significance is indicated as follows ****p < 0.0001, ***p < 0.001, **p < 0.01, and *p < 0.05.

To assess AHFV disease progression in IFNAR^-/-^ mice, we performed a detailed pathogenesis study for which we infected IFNAR^-/-^mice (*n* = 18) with 1000 LD_50_ (400 TCID_50_) by the IP route. Control mice were injected with DMEM. On 2 and 4 dpi, we euthanized six animals from AHFV-infected group. The remaining six mice were observed for body weight loss and survival (Figure 1(E,F)). The infection resulted in statistically significant haematology changes compared to control mice (Figure 2(A–F)). A low white blood cell (WBC) count on 2 and 4 dpi (Figure 2(A)) seemed to be largely caused by lymphopenia (Figure 2(B)) with contribution from eosinopenia (Figure 2(C)). Further abnormalities were noticed with reduced counts of reticulocytes (Figure 2(D)) and platelets (PLT) (Figure 2(E)) and an increase in neutrophils (Figure 2(F)). Various parameters of blood chemistry from AHFV-infected mice showed statistically significant deviations from control mice (Figure 2(G–L)). Infected mice had elevated levels of liver enzymes such as aspartate aminotransferase (AST) (Figure 2(G)) and alanine aminotransferase (ALT) (Figure 2(H)) as well as hypoglycemia (Figure 2(I)) on 2 dpi. By 4 dpi, partial recovery was observed in the levels of glucose, AST and ALT. Furthermore, hypoalbuminemia (Figure 2(J)), hyperglobulinemia (Figure 2(K)) and elevated levels of blood urea nitrogen (BUN) were observed on 4dpi (Figure 2(L)).

**Figure 3.**
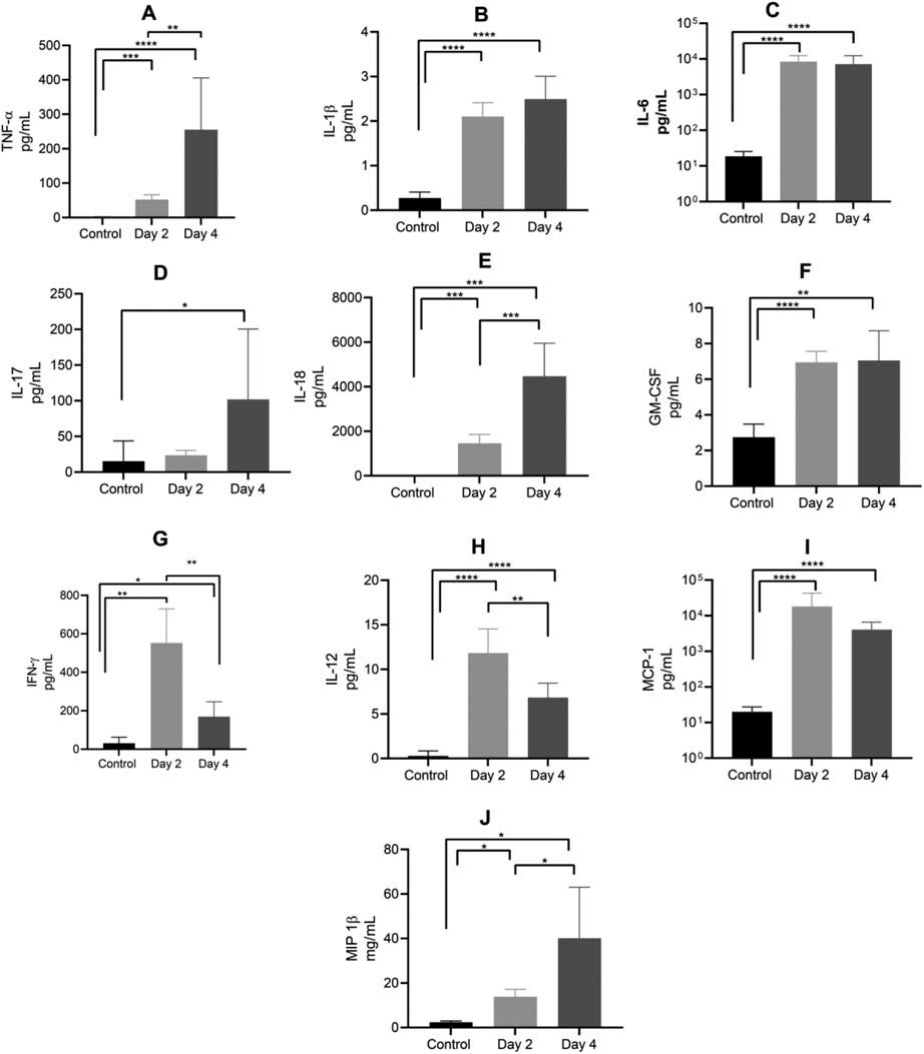
Plasma cytokine levels in mice infected with AHFV. Mice were infected IP with 1000 LD_50_ (400 TCID_50_). Animals were euthanized on 2 or 4 dpi and plasma samples were analyzed for cytokine expression in comparison to uninfected control mice. Results for TNF-*α*, IL-1*β*, IL-6, IL-17, IL-18, GM-CSF, IFN-γ, IL-12p40, MCP-1 and MIP-1*β* are shown. Error bars represent standard deviations. Statistical significance is indicated as follows ****p < 0.0001, ***p < 0.001, **p < 0.01, and *p < 0.05.

The expression of multiple proinflammatory cytokines was significantly elevated in plasma of AHFV-infected mice (Figure 3). TNF-*α*, IL-1*β*, IL-6, IL-17, IL-18, and GM-CSF levels were up on 2 dpi compared to the control mice with a further increase on 4 dpi (Figure 3(A–F), respectively). IFN-γ was highest on 2 dpi and returned to normal on 4 dpi (Figure 3(G)). Cytokines that promote macrophage recruitment such as IL-12p40, monocyte chemoattractant protein 1 (MCP-1) and macrophage inflammatory protein 1*β* (MIP-1*β*) were also elevated following AHFV infection (Figure 3(H–J)).

### AHFV infection of IFNAR^-/-^ mice showed a broad tissue tropism with restricted pathology

**Figure 4.**
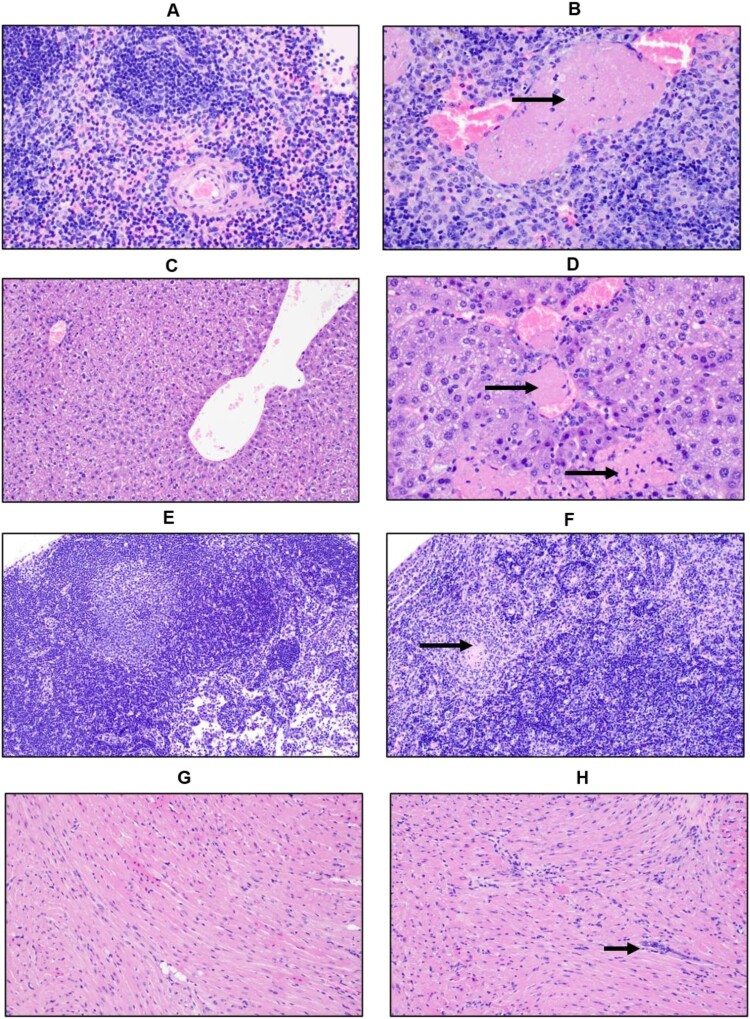
Histopathologic changes in IFNAR^-/-^ mice infected with AHFV. Groups of six mice were infected IP with 1000 LD_50_ (400 TCID_50_) and euthanized at 2 dpi and 4 dpi for organ harvest. (A and B) H&E stain of spleens from mice at 4 dpi (A, Mock 400×; B, AHFV infection 400×). We observed a reduction in the white pulp and expansion of the red pulp in infected animals indicating lymphoid depletion. There was neutrophil infiltration and vascular occlusion by a fibrin thrombus (arrow). (C and D) H&E stain of livers from mice at 4 dpi (C, Mock 200×; D, AHFV infection 400×). Hepatocytes were swollen and contained lipid micro-vesicles. There was an increase in sinusoidal inflammatory cells and, along the bottom of the frame, a focus of necrosis (bottom arrow). Fibrin thrombi occluded adjacent vessels (top arrow). (E and F) H&E stain of cervical lymph nodes from mice at 4 dpi (E, Mock 200×; F, AHFV infection 200×). Lymph nodes from infected animals exhibited tremendous lymphocyte apoptosis, infiltrating neutrophils and occasional fibrin thrombi (arrow). (G and H) H&E stain of heart from mice at 4 dpi (E, Mock 200×; F, AHFV infection 200×). Increased cellularity due to interstitial inflammation was seen in infected animals (arrows).

Infected animals had enlarged spleens, pale livers and about 50% of the mice had intestinal haemorrhages. Microscopic examination of the spleen revealed inflammation with neutrophils, lymphoid depletion of follicles (Figure 4(A,B)), apoptotic lymphocytes and vascular occlusion by fibrin thrombi. There were foci of coagulative necrosis within the liver of more than half of the mice (Figure 4(C,D)). Necrotic foci were often adjacent to blood vessels occluded by thrombi; however, the adjacent hepatic parenchyma appeared normal and neither the necrotic foci nor the adjacent hepatocytes are positive for AHFV suggesting necrotic foci are secondary to the presence of thrombi. The cervical lymph nodes contained viable and degenerate neutrophils and increased numbers of macrophages within sinuses at 2 dpi (Figure 4(E,F)). At 4 dpi, apoptotic lymphocytes were pervasive throughout the lymph nodes. The heart of some mice is infiltrated by mononuclear cells in the perivascular interstitium and between cardiac myocytes. The heart of all infected animals demonstrated mononuclear interstitial inflammation at 4 dpi (Figure 4(G,H)). No pathological abnormalities were observed in the brain, kidney or lungs (data not shown).

**Figure 5.**
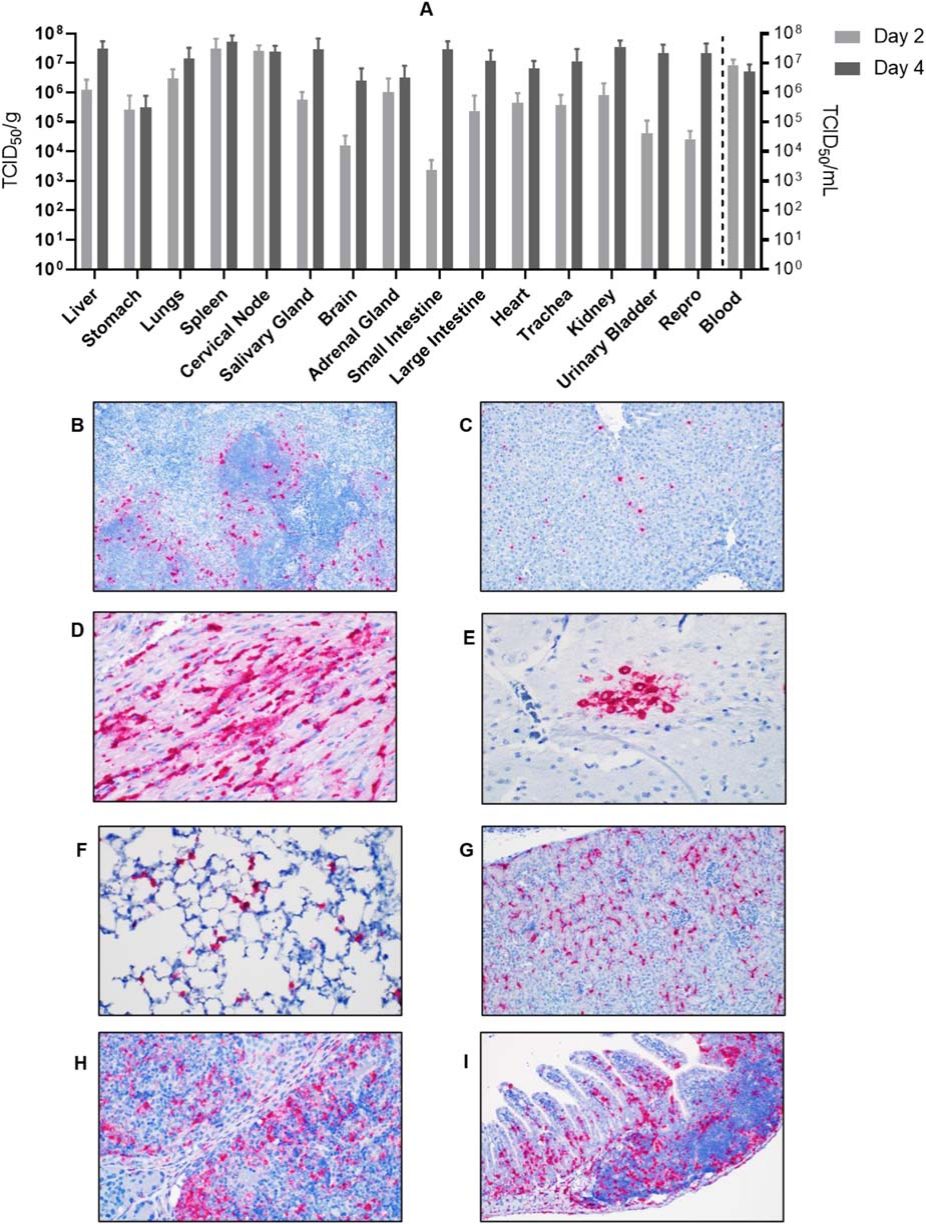
Tissue tropism of AHFV in infected mice. Groups of six mice were infected with 1000 LD_50_ (400 TCID_50_)/mouse via the IP route. The groups were euthanized on 2 and 4 dpi and organs were harvested to determine virus load. (A) The viral load in tissue is presented in TCID_50_/g; viremia is presented as TCID_50_/ml. (B–I) *In-situ* hybridization of AHFV genome in organs of infected mice. (B) Spleen 2 dpi; AHFV RNA was present at the marginal zone where the red and white pulp interact (200×); (C) Liver 4 dpi; AHFV RNA was located within individual mononuclear cells (Kupffer cells; 200×); (D) Heart 4 dpi; AHFV RNA corresponded with areas of inflammation (400×); (E) Brain 4 dpi; AHFV RNA was focally present within a cluster of neurons in the grey matter of the temporal lobe (400×); (F) Lungs 4 dpi; AHFV RNA was multifocally present throughout alveolar septa, presumably within macrophages (400×); (G) Kidney 4 dpi; AHFV RNA was detected within the interstitial tissue of the kidney (200×); (H) Cervical lymph node 4 dpi; AHFV RNA was located within mononuclear cells of the subcapsular sinuses and throughout lymphoid follicles (400×); (I) Small intestine, gut associated lymphoid tissue 4 dpi; AHFV RNA was present in GALT and lamina propria of intestinal villi (200×).

AHFV strain 2003 caused a systemic infection with high viremia (>10^7^ TCID_50_/ml) and viral load titers in all analyzed organs ranging from 10^3^ to almost 10^8^ TCID_50_/g tissue (Figure 5(A)). Viral titers were already high on 2 dpi but increased for most organs on 4 dpi (Figure 5(A)). There were no survivors under these conditions as noted above (Figure 1(F)). As expected from the organ viral loads, *in-situ* hybridization revealed AHFV dissemination in all organs analyzed (Figure 5(B–I)). In spleen, AHFV RNA was most prevalent in perifollicular marginal zones where the red and white pulp interact (Figure 5(B)). AHFV RNA in the liver was located within individual mononuclear cells (Kupffer cells) (Figure 5(C)) and in the heart, between cardiac myocytes (Figure 5(D)). In the brain, AHFV RNA was focally present within a cluster of neurons in the grey matter of the temporal lobe (Figure 5(E)). AHFV RNA was also detected within the septal macrophages of the lungs (Figure 5(F)), the interstitial tissue of the kidneys (Figure 5(G)), the subcapsular sinuses and the marginal zone of lymphoid follicles (Figure 5(H)), and the lamina propria of the intestinal villi (Figure 5(I)).

## Discussion

AHFV is a neglected zoonotic pathogen with potential impact on regional public and potentially animal health. The human AHF outbreaks in Saudi Arabia [12,28] and Egypt [29] as well as findings of AHFV-infected ticks on migratory birds [30] indicate the potential for AHFV emergence/re-emergence and a broadened geographic range [15]. Hence, enhanced awareness of AHF is warranted and the development of vaccines and therapeutics seems prudent. A major roadblock for countermeasure development is the lack of well-characterized AHFV animal disease model which is also needed for a better understanding of AHFV pathogenesis.

We here report a uniform lethal AHFV animal model utilizing immunocompromised IFNAR^-/-^ mice. This is in contrast to previous AHFV infection in commonly used immunocompetent laboratory mouse strains, which resulted in either no clinical disease (BALB/c) or 10%–50% lethality (C57BL/6, C3H and A/J) with surviving mice showing no or only mild clinical signs of disease [16,17]. This observation indicates the limitation of AHFV to antagonize murine IFN-*α*/*β* signalling or effector functions efficiently, analogous to other flavivirus such as ZIKV, YFV and DENV [31–33]. Immunocompromised mice, such as IFNAR^-/-^, are generally more susceptible to infections and these mice have been instrumental as disease models for a wider range of viral pathogens including ZIKV, DENV, YFV, WNV, CCHFV and Ebola virus [18–22,32–34]. With a low LD_50_ as demonstrated here for AHFV strain 2003 (Figure 1(D)), we have established a highly sensitive AHFV model that mimics severe cases of human AHFV infection. Hence, this model can provide a better understanding of AHFV pathogenesis and will be useful for countermeasure development against this emerging, neglected pathogenic flavivirus.

We compared two strains of AHFV, Zaki-2 and 2003. AHFV, strain Zaki-2 is the prototype strain isolated from a human in Jeddah, in 1994 [4], whereas AHFV, strain 2003 was isolated from a human from Makkah, Saudi Arabia, in 2003 [24]. The moderate increase in virulence of strain 2003 in mice (Figure 1) might be attributed to differences in the passage history with accumulation of mutations over passages. Amino acid sequence comparison between both strain Zaki-2 and strain 2003 revealed 13 amino acid changes (Supplementary Table) indicating the possibility of these mutations to alter protein function and thus change virulence. However, future experimental verification is required to confirm whether the accumulated mutations are associated with higher virulence.

AHFV-infected IFNAR^-/-^ mice developed haemorrhagic and neurologic disease manifestations, including intestinal bleeding, partial paralysis and tremors, as has been described for human AHF cases [5,13,35]. AHFV seeded an early viremia in these animals and spread and replicated systemically (Figures 4 and 5) similar to AHFV infection in humans [12,13]. AHFV distribution in IFNAR^-/-^ mice differed from that in immunocompetent mouse models, where the virus was only detected in certain organs such as kidneys, liver, and spleen at early infection time points. Thus, it seems that AHFV pathogenesis in IFNAR^-/-^ mice more closely reflects human AHF infections. Type-1 IFN has been shown to play a critical role in evading tick-borne encephalitis virus (TBEV) pathogenesis in mice [36–38], a situation that differs from the host response in IFNAR^-/-^ mice. Many published reports shed light on the evasion strategies of TBEV from the human IFN response [39,40]. One of them is passive evasion, which involves hiding viral dsRNA intermediates in vesicular structures of ER membranes that leads to delay in the recognition by the cytosolic RIG-I like receptors and subsequent IRF3 phosphorylation and IFN induction [41,42]. The other strategy is to antagonize IFNAR signalling through NS5-mediated inhibition of the JAK-STAT pathway [43,44]. In TBEV, cell surface expression of IFNAR1 is inhibited by NS5 by binding to prolidase, a peptidase that is needed for IFNAR1 maturation and subsequent cell surface expression [45]. Also, KFDV has been shown to resist IFN-mediated antiviral effects that involved NS5 [40]; nothing is known for AHFV.

AHFV patients regularly display blood cell abnormalities such as lymphopenia and thrombocytopenia, as well as blood chemistry abnormalities including elevated BUN, liver transaminases and hypoalbuminemia [12,13,15,35]. Our mouse model accurately mimics these pathophysiological parameters (Figure 2). Conversely, AHFV disease in our mouse model differed from published descriptions of human disease in certain other aspects. The creatinine levels were normal in infected mice suggesting normal kidney function. Also, no pneumonitis was seen in the lungs of infected mice. These outcomes might be attributed to the different routes of infection (IP in mice versus tick bite in humans) or genetic diversity among AHFV strains.

The cytokine induction in humans following AHFV infection has not been analyzed so far. Our results clearly demonstrate the inflammatory immune activation in AHFV-infected IFNAR^-/-^ mice (Figure 3). Previously, induction of inflammatory cytokines post infection was exclusively linked with antiviral effects. However, recent findings demonstrate that an overwhelming and imbalanced profile of cytokines could become excessive and harmful [46,47]. For example, the cytokine storm that follows severe primary DENV infection is mostly associated with the exacerbation of disease rather than protection against severe infection [48]. Chikungunya severity was linked with cytokines of IL-1*β*, IL-6, and RANTES produced in the acute phase [49]. AHFV infection in immunocompetent mice also resulted in the induction of proinflammatory cytokine responses [16]. A comparative study of AHFV infection in the non-lethal versus lethal mouse models could provide valuable insight into biomarkers for human AHFV infection.

Even though the virus replicated systemically, pathology was only observed in spleen, liver, heart and cervical lymph nodes; organs commonly targeted by AHFV infections in humans [4,8,12,13,15,35]. Extensive damage was seen in spleen and liver (Figure 4(A–D)) resulting in inflammatory infiltration by lymphocytes and macrophages, vascular occlusion by fibrin thrombi as well as necrotic lesions, consistent with profound hepatic and splenic disease seen in human AHF cases [6,12,14,15]. However, we did not notice any histologic abnormalities in brain tissue even though some mice displayed neurologic signs of disease and replicating AHFV was isolated from brain tissue (Figure 5(A)). Brain tissue ISH demonstrated a rather focal distribution mainly close to neurons in the grey matter of the temporal lobe (Figure 5(E)). Thus, tissue sampling may be an explanation for the discrepancy in our results. More detailed future studies are needed on the effect of AHFV replication in the central nervous system.

It is interesting to note that the related KFDV causes lethal disease in immunocompetent mice [16,17]. The infection is primarily neurological with the brain as the key site of KFDV replication. It has been speculated that KFDV may have acquired neuroinvasiveness during passaging in suckling mouse brain [16,17]. AHFV, the more recently discovered lower passage variant, still maintains its haemorrhagic phenotype. However, these speculations need to be confirmed experimentally by using a low passage KFDV variant. Noteworthy, KFDV infections have not yet been studied in IFNAR^-/-^ mice and, thus, a comparison with AHFV infection in an IFN-compromised environment cannot be done.

In conclusion, AHFV infection caused uniform lethal disease in IFNAR^-/-^ mice. The infection was followed by a rapid disease progression characterized by systemic replication with high viral loads in most organs, significant haematology and clinical chemistry abnormalities, systemic inflammatory immune response and marked pathology in liver and spleen. The infected mice succumbed to disease within 5–8 days after challenge. The disease in the IFNAR^-/-^ mouse model mimics severe human AHF [13–15]. This model will be helpful for the development of urgently needed countermeasures against this emerging neglected pathogen.

## Supplementary Material

Bhatia_et_al_Supplmentary_Material_Revision.docx
