# Supplementary material for: Alkhurma haemorrhagic fever virus causes lethal disease in IFNAR-/- mice
Source: Emerg Microbes Infect. 2021 Jun 6;10(1):1077–87. doi: 10.1080/22221751.2021.1932609 (PMC8183530; doi:10.1080/22221751.2021.1932609)
Supplement: Bhatia_et_al_Supplmentary_Material_Revision.docx [file TEMI_A_1932609_SM3991.docx]

**Supplementary Table: Amino acid comparison between AHFV strain Zaki-2 and AHFV strain 2003**

| **Gene** | **Position** | **Mutation** | **Function** |
| --- | --- | --- | --- |
| Capsid | 73 | A-V | Virus assembly and budding |
| prM | 227 | R-K | Prevents Envelope fusion during intracellular virion assembly |
| Env | 370 | S-G | Binds to host cell surface receptor and mediates fusion between viral and cellular membranes. |
| Env | 560 | S-N |  |
| Ns1 | 857 | S-N | Immune evasion, pathogenesis, and viral replication. |
| Ns2A | 1297 | A-T | Component of genome replication complex |
| NS3 | 2706 | K-R | Serine protease, NTPase and RNA helicase |
| Ns4A | 2215 | Y-H | Intracellular membrane rearrangement |
| NS5 | 2645 | V-A | RNA-directed RNA polymerase, methyl transferase and immune evasion |
| NS5 | 2765 | A-T |  |
| NS5 | 3157 | V-M |  |
| NS5 | 3212 | E-K |  |
| NS5 | 3342 | G-R |  |

Zaki_JF416957 MAKGAVLKGKGGGPPRRVPKETAKKTRQGPGRLPNGLVLMRMMGVLWHMIAGTARSPILK **Capsid**

2003_JF416954 MAKGAVLKGKGGGPPRRVPKETAKKTRQGPGRLPNGLVLMRMMGVLWHMIAGTARSPILK

************************************************************

**ER anchor for Capsid**

Zaki_JF416957 RFWATVPVRQAI**A**ALRKIRKTVGLLLDSLNRRRGKRRSTTGLLTSILLACLATLVISATI

2003_JF416954 RFWATVPVRQAI**V**ALRKIRKTVGLLLDSLNRRRGKRRSTTGLLTSILLACLATLVISATI

************.***********************************************

Zaki_JF416957 RRERTGDMVIRAEGKDAATQVEVVNGTCIILATDMGSWCDDSIMYECVTIDSGEEPVDVD **prM**

2003_JF416954 RRERTGDMVIRAEGKDAATQVEVVNGTCIILATDMGSWCDDSIMYECVTIDSGEEPVDVD

************************************************************

Zaki_JF416957 CFCRGVERVSLEYGRCGKPVGGRSRRSVSIPVHAHSDLTGRGHKWL**R**GDSVKTHLTRVEG

2003_JF416954 CFCRGVERVSLEYGRCGKPVGGRSRRSVSIPVHAHSDLTGRGHKWL**K**GDSVKTHLTRVEG

**********************************************:*************

Zaki_JF416957 WVWKNKLLTMAFCAVVWMVTDSLPTRFIVITVALCLAPTYATRCTHLQNRDFVSGIQGTT **Env**

2003_JF416954 WVWKNKLLTMAFCAVVWMVTDSLPTRFIVITVALCLAPTYATRCTHLQNRDFVSGIQGTT

************************************************************

Zaki_JF416957 RVSLVLELGGCVTLTAEGKPSVDVWLDDIHQENPAKTREYCLHAKLASSKVVARCPAMGP

2003_JF416954 RVSLVLELGGCVTLTAEGKPSVDVWLDDIHQENPAKTREYCLHAKLASSKVVARCPAMGP

************************************************************

Zaki_JF416957 ATLPEEHQA**S**TVCRRDQSDRGWGNHCGLFGKGSIVACAKFACEAKKKATGYVYDVNKITY

2003_JF416954 ATLPEEHQA**G**TVCRRDQSDRGWGNHCGLFGKGSIVACAKFACEAKKKATGYVYDVNKITY

*********.**************************************************

Zaki_JF416957 VVKVEPHTGDYLAANESHSNRKTASFTTQSEKTILTLGDYGDISLTCRVTSGVDPAQTVV

2003_JF416954 VVKVEPHTGDYLAANESHSNRKTASFTTQSEKTILTLGDYGDISLTCRVTSGVDPAQTVV

************************************************************

Zaki_JF416957 LELDKTAEHLPKAWQVHRDWFEDLSLPWRHEGAHEWNHADRLVEFGEPHAVKMDIFNLGD

2003_JF416954 LELDKTAEHLPKAWQVHRDWFEDLSLPWRHEGAHEWNHADRLVEFGEPHAVKMDIFNLGD

************************************************************

Zaki_JF416957 QTGILLKSLAGVPVANIEG**S**KYHLQSGHVTCDVGLEKLKMKGMTYTVCEGSKFAWKRPPT

2003_JF416954 QTGILLKSLAGVPVANIEG**N**KYHLQSGHVTCDVGLEKLKMKGMTYTVCEGSKFAWKRPPT

*******************.****************************************

Zaki_JF416957 DSGHDTVVMEVTYTGSKPCRIPVRAVAHGEPNVNVASLITPNPSMETTGGGFVELQLPPG

2003_JF416954 DSGHDTVVMEVTYTGSKPCRIPVRAVAHGEPNVNVASLITPNPSMETTGGGFVELQLPPG

************************************************************

Zaki_JF416957 DNIIYVGELSHQWFQKGSTIGRVLEKTRRGIERLTVVGEHAWDFGSVGGVLSSVGKALHT

2003_JF416954 DNIIYVGELSHQWFQKGSTIGRVLEKTRRGIERLTVVGEHAWDFGSVGGVLSSVGKALHT

************************************************************

Zaki_JF416957 AFGAAFNTIFGGVGFLPRILLGVALAWLGLNSRNPTLSVGFLITGGLVLTMTLGVGADMG

2003_JF416954 AFGAAFNTIFGGVGFLPRILLGVALAWLGLNSRNPTLSVGFLITGGLVLTMTLGVGADMG

************************************************************

Zaki_JF416957 CAIDANRMELRCGEGLVVWREVTDWYDGYAFHPESPPVLAASLKEAYEEGVCGIVPQNRL **Nsp1**

2003_JF416954 CAIDANRMELRCGEGLVVWREVTDWYDGYAFHPESPPVLAASLKEAYEEGVCGIVPQNRL

************************************************************

Zaki_JF416957 EMAMWRRVEAVLNLALAE**S**DANLTVVVDRRDPSDYRGGKVGILKRSGKEMKTSWKGWSQS

2003_JF416954 EMAMWRRVEAVLNLALAE**N**DANLTVVVDRRDPSDYRGGKVGILKRSGKEMKTSWKGWSQS

******************.*****************************************

Zaki_JF416957 FVWSVPESPRRFMVGIEGTGECPLDKRRTGVFTVAEFGMGMRTKIFLDLRETSSSDCDTG

2003_JF416954 FVWSVPESPRRFMVGIEGTGECPLDKRRTGVFTVAEFGMGMRTKIFLDLRETSSSDCDTG

************************************************************

Zaki_JF416957 VMGAAVKSGHAVHTDQSLWMKSHRNATGVFISELIVTDLRNCTWPASHTLDNAGVVDSKL

2003_JF416954 VMGAAVKSGHAVHTDQSLWMKSHRNATGVFISELIVTDLRNCTWPASHTLDNAGVVDSKL

************************************************************

Zaki_JF416957 FLPVSLAGPRSHYNHIPGYAEQVRGPWNQTPLRVVREPCPGTTVKIDQNCDKRGSSLRST

2003_JF416954 FLPVSLAGPRSHYNHIPGYAEQVRGPWNQTPLRVVREPCPGTTVKIDQNCDKRGSSLRST

************************************************************

Zaki_JF416957 TESGKAIPEWCCRTCELPPVTFRSGTDCWYAMEIRPVHQQGGLVRSMVLADNGAMLSEGG

2003_JF416954 TESGKAIPEWCCRTCELPPVTFRSGTDCWYAMEIRPVHQQGGLVRSMVLADNGAMLSEGG

************************************************************

Zaki_JF416957 VPGIVAVFVVLELVIRRRPTTGTSVVWCGVVVLGLVVTGLVTIEGLCRYVVAVGILMSME **Nsp2A**

2003_JF416954 VPGIVAVFVVLELVIRRRPTTGTSVVWCGVVVLGLVVTGLVTIEGLCRYVVAVGILMSME

************************************************************

Zaki_JF416957 LGPEIVALVLLQAVFDMRTGLLVAFAVKRAYTTREAVVTYFLLLVLELGFPEASLSNIWK

2003_JF416954 LGPEIVALVLLQAVFDMRTGLLVAFAVKRAYTTREAVVTYFLLLVLELGFPEASLSNIWK

************************************************************

Zaki_JF416957 WADSLAMGTLILQACSQEGRARVGYLLAAMMTQKDM**A**IIHTGLTIFLSAATAMAVWSMIK

2003_JF416954 WADSLAMGTLILQACSQEGRARVGYLLAAMMTQKDM**T**IIHTGLTIFLSAATAMAVWSMIK

************************************:***********************

Zaki_JF416957 GQRDQKGLSWATPLVGLFGGEGVGLRLLAFRRLAERRNRRSFSEPLTVVGVMLTVASGMV **NS2B**

2003_JF416954 GQRDQKGLSWATPLVGLFGGEGVGLRLLAFRRLAERRNRRSFSEPLTVVGVMLTVASGMV

************************************************************

Zaki_JF416957 RHTSQEALCALVAGAFLLLMMVLGTRKMQLIAEWCGEVEWNPDLVNEGGEVNLKVRQDAM

2003_JF416954 RHTSQEALCALVAGAFLLLMMVLGTRKMQLIAEWCGEVEWNPDLVNEGGEVNLKVRQDAM

************************************************************

Zaki_JF416957 GNLHLTEVEKEERAMALWLLAGLVASAFHWAGILIVLAIWTFFEMLSSGRRSELVFSGQG **NS3**

2003_JF416954 GNLHLTEVEKEERAMALWLLAGLVASAFHWAGILIVLAIWTFFEMLSSGRRSELVFSGQG

************************************************************

Zaki_JF416957 TRTERNRPFEIKDGAYRIYSPGLLWGHRQIGVGYGAKGVLHTMWHVTRGAALVVEEAISG

2003_JF416954 TRTERNRPFEIKDGAYRIYSPGLLWGHRQIGVGYGAKGVLHTMWHVTRGAALVVEEAISG

************************************************************

Zaki_JF416957 PYWADVREDVVCYGGAWSLESRWRGETVQVHAFPPGRPQETHQCQPGELILENGRKLGAV

2003_JF416954 PYWADVREDVVCYGGAWSLESRWRGETVQVHAFPPGRPQETHQCQPGELILENGRKLGAV

************************************************************

Zaki_JF416957 PIDLSKGTSGSPIINAQGEVVGLYGNGLKTNEAYVSSIAQGEAEKSRPELPLSVQGTGWM

2003_JF416954 PIDLSKGTSGSPIINAQGEVVGLYGNGLKTNEAYVSSIAQGEAEKSRPELPLSVQGTGWM

************************************************************

Zaki_JF416957 SKGQITVLDMHPGSGKTHRVLPELVRQCANRGMRTLVLAPTRVVLKEMEKALAGKKVRFH

2003_JF416954 SKGQITVLDMHPGSGKTHRVLPELVRQCANRGMRTLVLAPTRVVLKEMEKALAGKKVRFH

************************************************************

Zaki_JF416957 SPAVEGQSTAGAVVDVMCHATYVHRRLLPQGRQNWEVAIMDEAHWTDPHSIAARGHLYSL

2003_JF416954 SPAVEGQSTAGAVVDVMCHATYVHRRLLPQGRQNWEVAIMDEAHWTDPHSIAARGHLYSL

************************************************************

Zaki_JF416957 AKENRCALVLMTATPPGRGDPFPESNGAIMSEERAIPDGEWREGFDWITEYEGRTAWFVP

2003_JF416954 AKENRCALVLMTATPPGRGDPFPESNGAIMSEERAIPDGEWREGFDWITEYEGRTAWFVP

************************************************************

Zaki_JF416957 SISKGGAIARTLRQRGKSVICLNSKTFEKDYLRVREEKPDFVVTTDISEMGANLDVSRVI

2003_JF416954 SISKGGAIARTLRQRGKSVICLNSKTFEKDYLRVREEKPDFVVTTDISEMGANLDVSRVI

************************************************************

Zaki_JF416957 DGRTNIKPEEVDGKVEMTGTRKITTASAAQRRGRVGRTSGRTDEYIYSGQCDDDDTSLVQ

2003_JF416954 DGRTNIKPEEVDGKVEMTGTRKITTASAAQRRGRVGRTSGRTDEYIYSGQCDDDDTSLVQ

************************************************************

Zaki_JF416957 WKEAQILLDNITTLRGPVATFYGPEQMKMPEVAGHYRLNEEKRKHFRHLMTQCDFTPWLA

2003_JF416954 WKEAQILLDNITTLRGPVATFYGPEQMKMPEVAGHYRLNEEKRKHFRHLMTQCDFTPWLA

************************************************************

Zaki_JF416957 WHVATNTSNVLDRSWTWQGPEGNAIDGADGDLVRF**K**TPGGSERVLQPVWKDCRMFREGRD

2003_JF416954 WHVATNTSNVLDRSWTWQGPEGNAIDGADGDLVRF**R**TPGGSERVLQPVWKDCRMFREGRD

***********************************:************************

Zaki_JF416957 VKDFILYASGRRSVGDVLGGLAGVPGLLRHRCASALDVVYTLLNENPGSRAMRMAERDAP **Nsp 4A**

2003_JF416954 VKDFILYASGRRSVGDVLGGLAGVPGLLRHRCASALDVVYTLLNENPGSRAMRMAERDAP

************************************************************

Zaki_JF416957 EAFLTIVEVAVLGVATLGILWCFVARTSVSRMFLGTVVLFAALLLLWIGGVDYG**Y**MAGIA

2003_JF416954 EAFLTIVEVAVLGVATLGILWCFVARTSVSRMFLGTVVLFAALLLLWIGGVDYG**H**MAGIA

******************************************************:*****

**Peptide 2K**

Zaki_JF416957 LIFYIFLTVLQPEPGKQRSSDDNRLAYFLLGLLSLAGLVTANEMGMLDKTKADLAGLMWR **Nsp4B**

2003_JF416954 LIFYIFLTVLQPEPGKQRSSDDNRLAYFLLGLLSLAGLVTANEMGMLDKTKADLAGLMWR

************************************************************

Zaki_JF416957 GEQRHPAWEEWTNVDIQPARSWGTYVLIVSLFTPYMLHQLQTKIQQLVNSSVASGAQAMR

2003_JF416954 GEQRHPAWEEWTNVDIQPARSWGTYVLIVSLFTPYMLHQLQTKIQQLVNSSVASGAQAMR

************************************************************

Zaki_JF416957 DLGGGTPFFGVAGHVIALGVTSLVGATPLSLGLGVALAAFHLAIVASGLEAELTQRAHRV

2003_JF416954 DLGGGTPFFGVAGHVIALGVTSLVGATPLSLGLGVALAAFHLAIVASGLEAELTQRAHRV

************************************************************

Zaki_JF416957 FFSAMVKNPMVDGDVINPFPDGETKPVLYERRMSLILAIALCMVSVVLNRTAASMTEAGA

2003_JF416954 FFSAMVKNPMVDGDVINPFPDGETKPVLYERRMSLILAIALCMVSVVLNRTAASMTEAGA

************************************************************

Zaki_JF416957 VGLAALGQLVHPETETLWTMPMACGMAGLVRGSFWGLLPMGHRLWLKTTGTRRGGADGET

2003_JF416954 VGLAALGQLVHPETETLWTMPMACGMAGLVRGSFWGLLPMGHRLWLKTTGTRRGGADGET

************************************************************

Zaki_JF416957 LGDIWKRRLNGCSREEFFQYRRSGVMETERDRARELLKRGETNMGLAVSRGTAKLAWLEE **NS5**

2003_JF416954 LGDIWKRRLNGCSREEFFQYRRSGVMETERDRARELLKRGETNMGLAVSRGTAKLAWLEE

************************************************************

Zaki_JF416957 RGYATLKGEVVDLGCGRGGWSYYAASRPAVMGVKAYTIGGKGHEVPRLITSLGWNLIKFR

2003_JF416954 RGYATLKGEVVDLGCGRGGWSYYAASRPAVMGVKAYTIGGKGHEVPRLITSLGWNLIKFR

************************************************************

Zaki_JF416957 TGMD**V**YSLEAHRADTILCDIGESNPDPLVEGERSRRVILLMEKWKLRNPDASCVFKVLAP

2003_JF416954 TGMD**A**YSLEAHRADTILCDIGESNPDPLVEGERSRRVILLMEKWKLRNPDASCVFKVLAP

****.*******************************************************

Zaki_JF416957 YRPEVLEALHRFQLQWGGGLVRVPFSRNSTHEMYFSTAVSGNIVNSVNIQSRKLLARFGD

2003_JF416954 YRPEVLEALHRFQLQWGGGLVRVPFSRNSTHEMYFSTAVSGNIVNSVNIQSRKLLARFGD

************************************************************

Zaki_JF416957 QRGP**A**KVPEVDLGTGTRCVVLAEDKVREADVAERITALKTQYGDSWHVDKEHPYRTWQYW

2003_JF416954 QRGP**T**KVPEVDLGTGTRCVVLAEDKVREADVAERITALKTQYGDSWHVDKEHPYRTWQYW

****:*******************************************************

Zaki_JF416957 GSYKTEATGSAASLINGVVKLLSWPWNAREDVVRMAMTDTTAFGQQRVFKEKVDTKAQEP

2003_JF416954 GSYKTEATGSAASLINGVVKLLSWPWNAREDVVRMAMTDTTAFGQQRVFKEKVDTKAQEP

************************************************************

Zaki_JF416957 QVGTKIIMRAVNDWILERLAGKKTPRLCTREEFIAKVRSNAALGAWSDEQNRWSNAREAV

2003_JF416954 QVGTKIIMRAVNDWILERLAGKKTPRLCTREEFIAKVRSNAALGAWSDEQNRWSNAREAV

************************************************************

Zaki_JF416957 EDPEFWRLVDEERERHLRGRCAQCVYNMMGKREKKLGEFGVAKGSRAIWYMWLGSRYLEF

2003_JF416954 EDPEFWRLVDEERERHLRGRCAQCVYNMMGKREKKLGEFGVAKGSRAIWYMWLGSRYLEF

************************************************************

Zaki_JF416957 EALGFLNEDHWASRDLSGAGVEGISLNYLGWHLKRLSELEGGLFYADDTAGWDTRITNAD

2003_JF416954 EALGFLNEDHWASRDLSGAGVEGISLNYLGWHLKRLSELEGGLFYADDTAGWDTRITNAD

************************************************************

Zaki_JF416957 LEDEEQILRYLKGEHRTLAKTILEKAYHAKVVKVARPSSSGGCVMDIITRRDQRGSGQVV

2003_JF416954 LEDEEQILRYLKGEHRTLAKTILEKAYHAKVVKVARPSSSGGCVMDIITRRDQRGSGQVV

************************************************************

Zaki_JF416957 TYALNTLTNIKVQLIRMMEGEGVIGPSDSQDPRLLR**V**EAWLKEYGEERLTRMLVSGDDCV

2003_JF416954 TYALNTLTNIKVQLIRMMEGEGVIGPSDSQDPRLLR**M**EAWLKEYGEERLTRMLVSGDDCV

************************************:***********************

Zaki_JF416957 VRPIDDRFGKALYFLNDMAKVRKDIGEWEPS**E**GYSSWEEVPFCSHHFHELTMKDGRVIIV

2003_JF416954 VRPIDDRFGKALYFLNDMAKVRKDIGEWEPS**K**GYSSWEEVPFCSHHFHELTMKDGRVIIV

*******************************:****************************

Zaki_JF416957 PCRDQDELVGRARVSPGCGWSVRETACLSKAYGQMWLLSYFHRRDLRTLGLAICSAVPID

2003_JF416954 PCRDQDELVGRARVSPGCGWSVRETACLSKAYGQMWLLSYFHRRDLRTLGLAICSAVPID

************************************************************

Zaki_JF416957 WVPQGRTTWSIHASGAWMTTEDMLEVWNRVWILDNPFMSDK**G**KVKEWRDIPYLPKSQDGL

2003_JF416954 WVPQGRTTWSIHASGAWMTTEDMLEVWNRVWILDNPFMSDK**R**KVKEWRDIPYLPKSQDGL

***************************************** ******************

Zaki_JF416957 CSSLVGRRERAEWAKNIWGSVEKVRRMIGPERYADYLSCMDRHELHWDLKLESNII

2003_JF416954 CSSLVGRRERAEWAKNIWGSVEKVRRMIGPERYADYLSCMDRHELHWDLKLESNII

********************************************************
